# Dietary supplementation of *Allium mongolicum* modulates rumen-hindgut microbial community structure in Simmental calves

**DOI:** 10.3389/fmicb.2023.1174740

**Published:** 2023-06-07

**Authors:** Kaili Xie, Shenghua Chang, Jiao Ning, Yarong Guo, Cheng Zhang, Tianhai Yan, Fujiang Hou

**Affiliations:** ^1^State Key Laboratory of Grassland Agro-Ecosystems, Key Laboratory of Grassland Livestock Industry Innovation, Ministry of Agriculture, College of Pastoral Agriculture Science and Technology, Lanzhou University, Lanzhou, Gansu, China; ^2^Agri-Food and Biosciences Institute, Hillsborough, United Kingdom

**Keywords:** functional native herbage, community, gastrointestinal, ruminant, microorganism

## Abstract

Compared to traditional herbage, functional native herbage is playing more important role in ruminant agriculture through improving digestion, metabolism and health of livestock; however, their effects on rumen microbial communities and hindgut fermentation are still not well understood. The objective of present study was to evaluate the effects of dietary addition of *Allium mongolicum* on bacterial communities in rumen and feces of claves. Sixteen 7-month-old male calves were randomly divided into four groups (*n* = 4). All calves were fed a basal ration containing roughage (alfalfa and oats) and mixed concentrate in a ratio of 60:40 on dry matter basis. In each group, the basal ration was supplemented with *Allium mongolicum* 0 (SL0), 200 (SL200), 400 (SL400), and 800 (SL800) mg/kg BW. The experiment lasted for 58 days. Rumen fluid and feces in rectum were collected, Rumen fluid and hindgut fecal were collected for analyzing bacterial community. In the rumen, Compared with SL0, there was a greater relative abundance of phylum Proteobacteria (*p* < 0.05) and genera *Rikenellaceae_RC9_gut_group* (*p* < 0.01) in SL800 treatment. In hindgut, compared with SL0, supplementation of *A. mongolicum* (SL200, SL400, or SL800) decreased in the relative abundances of *Ruminococcaceae_UCG-014* (*p* < 0.01), *Ruminiclostridium_5* (*p* < 0.01), *Eubacterium_coprostanoligenes_group* (*p* < 0.05), and *Alistipes* (*p* < 0.05) in feces; Whereas, the relative abundances of *Christensenellaceae_R-7_group* (*p* < 0.05), and *Prevotella_1* (*p* < 0.01) in SL800 were higher in feces, to maintain hindgut stability. This study provided evidence that *A. mongolicum* affects the gastrointestinal of calves, by influencing microbiota in their rumen and feces.

## 1. Introduction

Some plants (e.g., *Gentiana straminea*, *Ligularia virgaurea*, and *Cistanche deserticola*) are often not favored by herbivores because of their unique taste or poor palatability (Liu X. et al., 2020; Xie et al., 2022; Cui et al., 2023). These undesirable plants are abundant all year round and thus can be used as non-conventional feed resources or “functional native herbage (FNH).” The whole plant or some organs of FNH are rich in secondary metabolites throughout the whole year or during the growing season. Compared with traditional herbage, FNH are rarely ingest by livestock. It is usually intake during non-growing season, or unavoidably intake by grazing livestock, or only actively intake in exceptional circumstances such as injury or illness (Wang et al., 2022). FNH can regulate digestion and metabolism, productivity and health of livestock. It plays an irreplaceable role in livestock production (Zhang et al., 2022). FNH comprises 16.8–26.8 and 13.5% of plant species and biomass in global terrestrial ecosystems, respectively (Richards et al., 2015; Jamshidi-Kia et al., 2018; Hou et al., 2021). The FNH contributes up to 15% of the livestock feed from grazing (Zhang and Zhang, 2017). Feeding amount and function of FNH are similar to fresh grass and additives, respectively (Harris et al., 1968). Multiple secondary metabolites (e.g., phenylethanoid glycoside, volatile components, iridoids, flavonoids, alkaloids, simple secoiridoid glycoside, flavonoids, and iridoid glycosides) in *Cistanche deserticola* and *Gentiana straminea* can directly alter enteric microbe involved in livestock digestion and metabolism (Liu X. et al., 2020; Xie et al., 2022).

Rumen bacterial community play a vital role in maintaining the normal digestion and absorption in the host (Liu et al., 2016). The bacterial community structure in feces plays an important role in fermentation of carbohydrates and proteins; and the composition of bacterial population is influenced by the chemical composition of diet (Petri et al., 2019). Although FNH was used as safe and effective feed additive in ruminant agriculture (Ahmad et al., 2020), and the effects of supplementing extracts from FNHs on the rumen microbiome have been studied; the effect of whole plant FNHs have not been thoroughly evaluated. Therefore, it is important from the ruminant nutritionists to gain a better understanding of the relationship between the rumen and fecal microbes and the potential contribution of FNH to gut nutrient metabolism and function.

**Figure 1:**
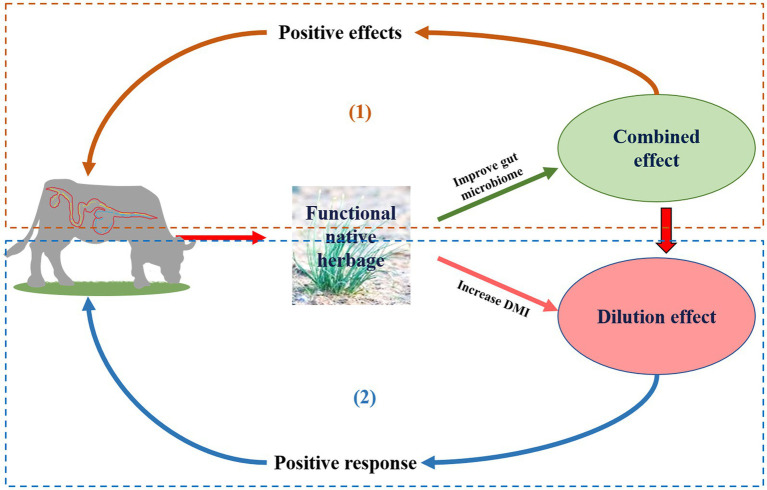
Conceptual sketch of effect of the whole functional native herbage (FNH) feed on gastrointestinal microorganisms of ruminants. (1) Combined effect: Multiple secondary metabolites in FNH have a positive effect on animals by combining to simultaneously produce beneficial effects on gastrointestinal microbes; (2) Dilution effect: Indirectly by promoting intake of other diets by ingesting FNH.

The rumen and hindgut microbial communities play an important role in the degradation of plant fiber and protein absorption (Cancino-Padilla et al., 2021), and their subsequent conversion into volatile fatty acids (VFA; Wang et al., 2016; Toral et al., 2018). Microbial fermentation of carbohydrates accounts for 5–10% of the total-tract digestibility of carbohydrates in the entire alimentary canal of cattle (Gressley et al., 2011). In previous studies (Panthee et al., 2017; Redoy et al., 2020), the effects of allicin, garlic powder, garlic oil, onion, and *Allium sativum* extracts have been examined on rumen fermentation and microbes *in vitro*. However, the effects of these additives have not been studied on gastrointestinal microbes in other parts of the digestive tract. Some secondary metabolites (condensed tannins, saponins, essential oils, and flavonoids) of FNHs, (*Cichorium L.*, *Allium L*., *Gentiana L*., and *Morus L*.), have been proved to improved the intestinal absorption of nutrients (Patra et al., 2017; Ibrahim et al., 2021). *Gentiana straminea* and *Ligularia virgaurea* contain many secondary metabolites, including monoterpenoids, diterpenoids, triterpenes, sesquiterpenes, simple secoiridoid glycoside, flavonoids, and iridoid glycosides, which may have antagonistic or synergistic on livestock performance and gastrointestinal flora (Xie et al., 2022; Cui et al., 2023), the whole herbage additions could enhance livestock performance and rumen microbes simultaneously. The “combined effect” consists of three aspects: firstly, a FNH normally contains a variety of active components which have a kind of synergistic or antagonistic effect on livestock performance as a wholeness; secondly, there were differences in the types and contents of various FNH and active components in diets, which had positive, negative or no effect on livestock; finally, the productive performance of livestock responds to the active component of FNH, which is reflected in the regulation of immune performance, digestion and metabolism, and growth performance, etc. The “dilution effect” consists of three aspects: firstly, due to the active component or the special odor of FNH, FNH increases or decreases the proportion of FNH in dry matter intake (DMI) by influencing DMI of livestock base diets; secondly, the bright color or active components of FNH regulate neuroendocrine feeding centers, improve the selective feeding of livestock, promote feed intake of forage with high water content (based on the some dry matter), stimulate gastric secretion, and reduce the concentration of FNH or its active components in internal organs and tissues; finally, The “combined effects” of multiple FNH_S_, or between FNH_S_ and diet, cause the active component to break down and reduce the concentration in the body. As a result, the whole herbage of FNH also has the potential to reduce methane emissions through a “combined effect” of multiple secondary metabolites (Nobler et al., 2019; Xie et al., 2020), or indirectly by promoting intake of other diets by ingesting whole forage. This “dilution effect” of multiple secondary metabolites also has a positive effect on livestock performance and the hindgut microbiome (Neave et al., 2018). Consequently, a comprehensive analysis of whole forage on ruminants’ microbiota is crucial for a realistic comparison of the differences in secondary metabolism. Therefore, it was hypothesized that whole herbage had a “combined effect” or “dilution effect” on the gastrointestinal microbes of ruminants (Figure 1).

*Allium,* a worldwide genus belonging to FNH, contains garlic oil, flavonoids, polysaccharides, alkaloids, etc. (Wingard et al., 2011), which are fed to ruminant livestock (Wanapat et al., 2015). *Allium mongolicum* grows widely in the Euro-Asia steppe zone (Huang et al., 2011), which is one of the most expansive nomadic regions of the world (Jeong et al., 2020). Despite *A. mongolicum* has potential to improve growth performance, nutrient digestibility, immunity, and rumen fermentation parameters of Simmental calf (Patra et al., 2017; Xie et al., 2020), the effects of whole plant feed supplements on the gastrointestinal microbiome of ruminants are not well understood. The present study was designed to evaluate the effect of *A. mongolicum* on the relationship of the microbes between the rumen and feces, to identify the relationship between intestinal function and rumen-hindgut microbes changes, and to provide evidence for the application of *A.mongolicum* on the diet of Simmental calf.

## 2. Materials and methods

### 2.1. Study site

The experiment was conducted at the Linze Pastoral Agriculture Station of Lanzhou University, in Linze County, Gansu Province, China (39.24°N, 100.06°E) from July to September 2017. The area is classified as a temperate arid, with an average annual temperature and precipitation of 7.7°C and 117.2 mm, respectively. The dominant agricultural systems here are intensively and extensively specialized crop and livestock production systems (Hou et al., 2021).

### 2.2. Feeding and management of animals

Sixteen 7-month-olds male Simmental calves (175 ± 10 kg), were randomly divided into four groups with four calves in each group. All calves were fed a basal ration containing roughage (alfalfa and oats) and mixed concentrate in a ratio of 60:40 on dry matter basis. The basal diet fulfilled the maintenance and growth requirements of calves to support 1 kg daily gain for fattening cattle (175 kg BW) according to the Chinese recommended beef cattle breeding standards (NY/T815-2004). In each group, the basal ration was supplemented with *A. mongolicum* 0 (SL0), 200 (SL200), 400 (SL400), or 800 mg/kg BW (SL800). The experiment lasted for 58 days. The nutrients of fresh *A. mongolicum* and basal diets (oat and alfalfa hay, soybean meal, wheat barn, and corn kernel) were determined (Table 1). Roughage was fed separately twice daily in equal amounts at 7 a.m. and 7 p.m. Fresh *A. mongolicum* was mixed with concentrates and offered at 2 pm. daily. All the calves were kept in single-pens (1.6 m × 3.6 m) and had free access to drinking water around the clock. A detailed description of the animal digestion and metabolism trial, laboratory analyses and calculation of DMI, body weight (BW), nutrient digestibility, fecal nutrient content, methane (CH_4_), volatile fatty acids (acetate, propionate, butyrate, and valerate) can be found in a complementary paper on Simmental claves (Xie et al., 2020).

**Table 1:** Chemical composition of the basal diets and *Allium mongolicum* Regel (% of DM).

Nutrient levels (% on DM basis)	Basal diets	*Allium mongolicum* Regel (%)
DM	87.9	6.3–17.1
OM	90.0	82.7
CP	14.9	25.8
NDF	46.6	24.8
EE	1.6	4.4

### 2.3. Fecal collection

Samples from rumen fluid and hindgut feces were taken on the last day of the experiment for analyze bacterial community. Fecal samples were collected and divided into three parts: one portion was dried at 65°C for 96 h to determine the neutral detergent fiber (NDF), acid detergent fiber (ADF), organic matter (OM) and ether extract (EE) content; other portion of fresh feces (100 g) was combined with 10 mL of 10% (v/v) sulfuric acid for N fixation and frozen at −20°C; and the final portion of the feces was placed in a vacuum tube and immediately stored in liquid nitrogen for bacterial determination.

Fecal collection, for subsequent analysis of its microbiota, was done following the method described by Petri et al. (2019). Specifically, fecal samples were collected from rectum of the calves by hand, using a new palpation sleeve for each calf at the 58 day of the experiment, between 600 to 630 h. The fecal sample collection and rumen collection were on the same day to reduce the stress of animals. The use of rectally collected fecal samples, for assessing hindgut fermentation profiles and microbial activity has been considered a reasonable approach (Gressley et al., 2011; Castillo-Lopez et al., 2020).

### 2.4. Rumen fluid sampling

Rumen fluid samples of 16 calves were taken via the orogastric tube technique on day 58. Two hours after morning feeding, a ruminal fluid collection tube (Anscitech Co., Ltd. Winnipeg, Manitoba, Canada) was inserted into the rumen and the rumen fluid was aspirated using a 150 mL syringe. After cleaning the orogastric tube with clean water, 100 mL of rumen fluid was discarded, preventing saliva contamination (Fan et al., 2021). The rumen fluid was collected in cryovials after filtration through double layers of gauze and immediately flash frozen in liquid nitrogen for subsequent bacterial analysis.

### 2.5. DNA isolation, 16S rRNA sequencing, and bioinformatics analyses

Genomic DNA was extracted from rumen fluid and feces by hexadecyl trimethyl ammonium Bromide (Minas et al., 2011). Purity and concentration of DNA was assessed by atomic spectrophotometry and 1% agarose gel electrophoresis. An appropriate amount of sample was aliquoted into a centrifuge tubes, and diluted to 1 ng/μL with sterile water. The V3-V4 hypervariable region of the bacterial 16S rRNA gene was amplified using universal primers forward primer (515F) 5′ -GTGCCAGCMGCCGCGG-3′ and reverse primer (806R) 5′-GGACTACHVGGGTWTCTAAT-3′. The PCR amplification reaction cycles for subsequent high-throughput sequencing were as follows: initial denaturation was done at 95°C for 2 min, followed by 30 cycles of 30 s at 95°C, 30 s at 55°C, and 60 s at 72°C, with a final extension at 72°C for 5 min. The PCR products were purified using 2% agarose gel electrophoresis. The TruSeq^®^ DNA PCR-Free Sample Preparation Kit was used for library construction. The constructed library was quantified using a Qubit and q-PCR. After qualification of the library, HiSeq2500 PE250 was used for on-machine sequencing. The 16-rumen fluid and 16 fecal samples were sequenced. Paired-end reads were assigned to samples based on their unique barcodes. The reads of each sample were spliced using FLASH (V1.2.7; Magoč and Steven, 2011) after truncating the barcode and primer sequences, and the splicing sequence obtained was considered the original Tag data (Raw Tags).^1^ The spliced Raw Tags underwent strict filtering for obtaining high-quality Tag data (Clean Tags; Bokulich et al., 2013). Low-quality raw tags were filtered using QIIME (V1.7.0; Caporaso et al., 2010).^2^ The tag sequence (UCHIME Algorithm)^3^ were compared with the database (Gold database)^4^ for detecting the chimera sequence, which were then removed for subsequent analyses (Effective Tags; Edgar et al., 2011; Haas et al., 2021). Sequence analyses was clustered by UPARSE into operational taxonomic units (OTUs) based on 97% similarity (Edgar, 2013). Representative sequences of OTUs were subjected to species annotation analysis using the SSUrRNA database of SILVA (Wang et al., 2007). Alpha diversity analysis for Observed-species, Chao1, Shannon, Simpson, and Goods-coverage indices was performed using the QIIME software (Version 1.7.0). The principal coordinates analysis (PCoA) was analyzed done using R software’s WGCNA, stats and ggplot2 packages using UniFrac distance-based principal coordinate analysis.

### 2.6. Statistical analysis

The data were statistically analyzed Normal distributions were checked using the Shapiro–Wilk test, using one-way analysis of variance using ANOVA procedures of SAS (SAS v 9.2, SAS Institute Inc., Cary, NC, United States). Treatment means were compared using LSD test and differences were considered statistically significant at *p* < 0.05.

Spearman correlation coefficients between chemical composition of herbage, nutrient digestibility, CH_4_, bacterial genus, and ruminal fermentation parameters was performed using R software (version 4.0.2).^5^ Microbial networks were generated to calculate the correlations between predominant taxa using Gephi software (version 0.9.2).^6^ The putative drivers of keystone taxa in the rumen and feces microbial communities of Simmental calves from the gastrointestinal region were estimated using combined score of the high mean degree, high closeness centrality, and low betweenness centrality. Spearman correlation coefficients between the relative abundances of the rumen bacteria (genus) and nutrient digestibility were calculated using the heatmap package in R software (version 4.0.2, see text footnote 5). The R.4.1.2 (Gemplus software package) was used to perform Aggregate Boosted Tree Analysis (ABT) to quantitatively analyze the correlations of nutrient digestibility, rumen fermentation parameters and fecal nutrient nutrients, and dominant bacteria in rumen and feces. Redundancy analysis (RDA) in CANOCO (Windows 4.5) was used to analyze the correlations of nutrient digestibility, rumen fermentation parameters, fecal nutrient nutrients, and dominant bacteria in rumen and feces.

## 3. Results

### 3.1. Microbial community richness, diversity, and distribution in rumen and feces

**Figure 2:**
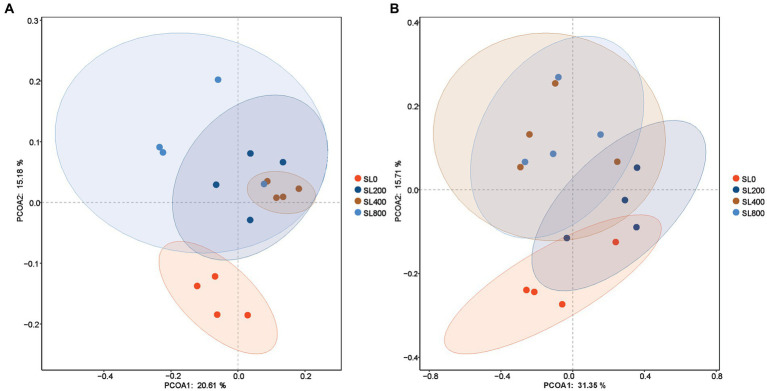
Alpha-diversity of rumen and fecal microbiome in Simmental calves fed different levels of *A. mongolicum*. **(A)** Rumen; **(B)** feces. SL0, 0 mg/kg BW *A. mongolicum*; SL200, 200 mg/kg BW *A. mongolicum*; SL400, 400 mg/kg BW *A. mongolicum*; SL800, 800 mg/kg BW *A. mongolicum*.

Chao 1 index in SL200 was significantly higher than that in SL0 (*p* = 0.036). The Goods_coverage value of the rumen and feces were 99.3 ± 0.2 and 99.5 ± 0.1%, respectively (Table 2). In feces, the bacterial species on the SL200 treatment was the lowest of all the treatment groups (*p* = 0.031). The Shannon and Simpson diversity indices of rumen and feces were not significantly different among the four treatment groups. In the rumen, each of the four treatments had a cluster of microorganisms (Figure 2A). However, the principal coordinate analysis showed clear separations of fecal bacteriome in SL0 and SL200 treatments. The observed spatial heterogeneity of the fecal bacterial community was similar in SL400 and SL800 treatment groups (Figure 2B).

**Table 2:** Alpha-diversity of the microbiome of rumen and fecal sample of calves fed different amounts of *A. mongolicum.*

Regions	Indexes	Treatments				SEM	*p*-value
		SL0	SL200	SL400	SL800		
Rumen	Observed_species	1656.33	1765	1631.75	1740.75	23.518	0.116
	Shannon	8.00^ab^	8.00^ab^	7.86^b^	8.02^a^	0.059	0.112
	Simpson	0.978	0.98	0.977	0.98	0.002	0.43
	Chao1	1762.3^b^	1962.68^a^	1788.73^ab^	1913.31^ab^	31.005	0.036
	Goods_coverage	0.995^a^	0.993^b^	0.994^ab^	0.993^b^	0.0002	0.029
Feces	Observed_species	1572.67^a^	1381.75^b^	1613.75^a^	1580^a^	34.026	0.031
	Shannon	7.56	6.03	7.28	7.34	0.265	0.149
	Simpson	0.956	0.926	0.955	0.952	0.0104	0.781
	Chao1	1721.05	1543.76	1743.76	1723.58	33.858	0.109
	Goods_coverage	0.994	0.994	0.995	0.995	0.001	0.754

SL0, 0 mg/kg BW *A. mongolicum*; SL200, 200 mg/kg BW *A. mongolicum*; SL400, 400 mg/kg BW *A. mongolicum*; SL800, 800 mg/kg BW *A. mongolicum*.

^a,b^ Different letters represent significant differences in the rows (*p* < 0.05); SEM, standard error of mean.

### 3.2. Bacterial community composition in the rumen and feces of Simmental calves

**Figure 3:**
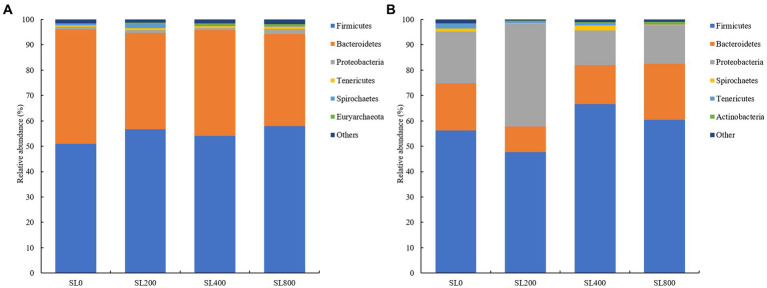
Composition of bacterial communities in Simmental calves at phylum levels in rumen **(A)** and feces [**(B)**; only taxa with an average relative abundance >0.5% are shown]. SL0, 0 mg/kg BW *A. mongolicum*; SL200, 200 mg/kg BW *A. mongolicum*; SL400, 400 mg/kg BW *A. mongolicum*; SL800, 800 mg/kg BW *A. mongolicum*.

There were 26 and 24 bacterial phyla observed in the rumen and fecal microbiota, respectively (Figure 3). At the phylum level, the relative abundance of the two predominant phyla Firmicutes and Bacteroidetes in rumen accounted for 52.9 and 40.3%, respectively (Figure 3A). Compared to SL0 treatment, the relative abundance of Proteobacteria were higher and of Bacteroidetes was lower (*p* < 0.05) in the SL800 treatment (Supplementary Table S1) in rumen.

In feces, Firmicutes (57.7% ± 4.4), Proteobacteria (22.5% ± 5.8), and Bacteroidetes (16. 6% ± 2.1) were the predominant bacteria, accounting for more than 96.7% of the total bacteria (Figure 3B). The relative abundances of Firmicutes (*p* < 0.01) were higher in the SL400 and SL800 than those in the SL0 treatment. The relative abundances of Spirochaetes (*p* < 0.01) were lower in the SL200 and SL800 than those in the SL0 and SL400 treatment groups (Supplementary Table S2) in feces. Relative abundance of Tenericutes (*p* < 0.05) was lower in the groups receiving *Allium mongolicum* than SL0 in feces. The relative abundances of Actinobacteria (*p* < 0.05) were higher in the SL400 than those in the SL0 treatment group. The ratio of Firmicutes to Bacteroidetes (*p* > 0.05) was not significantly different among the four treatment groups.

**Figure 4:**
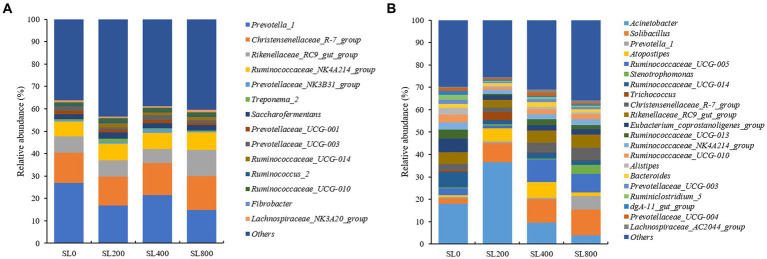
Composition of bacterial communities in Simmental calves at genus levels in rumen **(A)** and feces [**(B)**; only taxa with an average relative abundance >0.5% are shown]. SL0, 0 mg/kg BW *A. mongolicum*; SL200, 200 mg/kg BW *A. mongolicum*; SL400, 400 mg/kg BW *A. mongolicum*; SL800, 800 mg/kg BW *A. mongolicum*.

At the genus level, the relative abundance of *Prevotella_1* was the most dominant in ruminal fluid, followed by the *Christensenellaceae_R-7_group, Rikenellaceae_RC9_gut_group,* and *Ruminococcaceae_NK4A214_group* (Figure 4A). Compared with SL0, the relative abundances of *Prevotella_1* was lower (*p* < 0.05), representing 16.8 and 14.7% of the populations in the SL200 and SL800 groups, respectively (Supplementary Table S1). The relative abundance of *Rikenellaceae_RC9_gut_group* of the SL800 treatment was higher (*p* < 0.01) than in the SL0 and SL200 group, while SL400 was not significantly different than SL0, SL200, and SL800.

In feces, *Acinetobacter*, *Solibacillus*, and *Ruminococcaceae_UCG-005* were the dominant genera (Figure 4B). The relative abundances of *Prevotella_1* (*p* < 0.01), *Stenotrophomonas* (*p* < 0.01), and *Christensenellaceae_R-7_group* (*p* < 0.05) were significantly higher in the SL800 group than the SL0 group (Supplementary Table S2)*. Ruminococcaceae_UCG-005* (*p* < 0.05) had the greatest relative abundance in the SL400 group. The relative abundances of *Ruminococcaceae_UCG-014* (*p* < 0.01), *Ruminiclostridium_5* (*p* < 0.01), *Alistipes* (*p* < 0.05), *Eubacterium_coprostanoligenes_group* (*p* < 0.05), and *dgA-11_gut_group* (*p* < 0.01) were lower in feces of claves supplemented with *Allium Mongolicum* (at any rate) than observed in SL0. The highest relative abundance of *Trichococcus* (*p* < 0.01) was greatest in animals receiving SL200, while *Acinetobacter* (*p* < 0.05) was in the lowest abundance in feces of claves receiving SL400 and SL800 treatments.

The relative abundances of *Ruminiclostridium_5*, *Alistipes*, and *dgA-11_gut_group* had highest variation in the regions (*p* < 0.001) in feces of calves given different supplementation of *A. mongolicum* (*p* < 0.05) and supplementary level and regions interaction (*p* < 0.05; Supplementary Table S3). The relative abundances of *Acinetobacter* (*p* < 0.01), *Christensenellaceae_R-7_group* (*p* < 0.001), *Solibacillus* (*p* < 0.05), *Ruminococcaceae_UCG-010* (*p* < 0.01), *Prevotellaceae_NK3B31_group* (*p* < 0.01), *Saccharofermentans* (*p* < 0.001), *Prevotellaceae_UCG-001* (*p* < 0.001), *Fibrobacter* (*p* < 0.001), *Lachnospiraceae_NK3A20_group* (*p* < 0.001), *Ruminococcaceae_UCG-013* (*p* < 0.001), *Ruminococcaceae_NK4A214_group* (*p* < 0.001), *Bacteroides* (*p* < 0.001), and *Prevotellaceae_UCG-004* (*p* < 0.001) were affected by the gastrointestinal region.

### 3.3. The linkage between dry matter intake, nutrient digestibility, total VFA, methane, fecal nutrient, and rumen and feces microbial community

**Figure 5:**
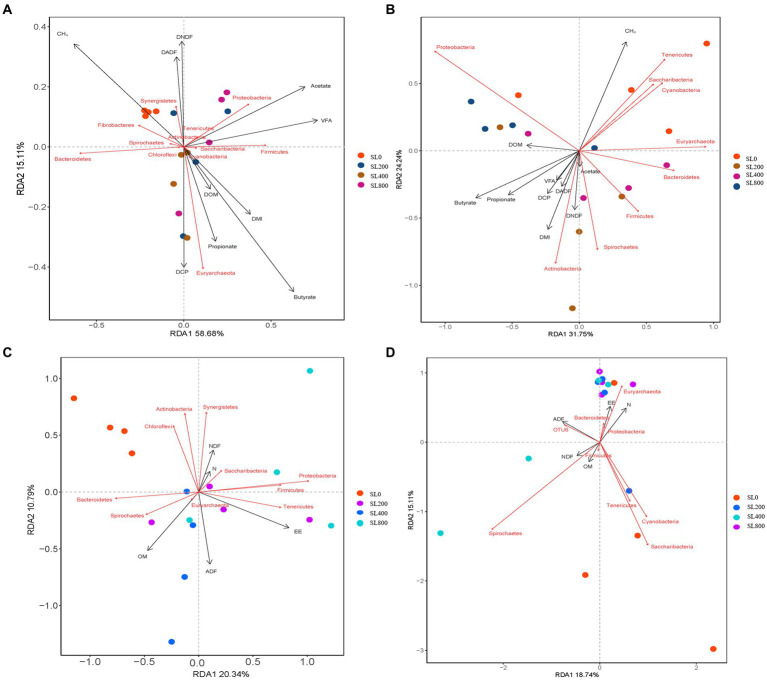
Redundancy analysis (RDA) for nutrient digestibility, rumen fermentation parameters, and fecal nutrients (black arrows) and rumen and fecal main bacterial (red arrows) phyla at varying supplement level of *A. mongolicum*. The length of the arrow indicates the correlation between nutrient digestibility, rumen fermentation parameters, fecal nutrients, and rumen or fecal main bacterial with the distribution of samples at different supplemental levels of *A. mongolicum*; the longer the arrow length, the greater the correlation. The direction of the arrow indicates trends in nutrient digestibility, rumen fermentation parameters, fecal nutrients, and major bacteria in the rumen or fecal. **(A)** Relations between rumen bacterial taxa (phylum) and nutrient digestibility and rumen fermentation parameters. **(B)** Relations between rumen bacterial taxa (phylum) and fecal nutrients. **(C)** Relations between fecal bacterial taxa (phylum) and nutrient digestibility and rumen fermentation parameters. **(D)** Relations between fecal bacterial taxa (phylum) and fecal nutrients. DMI, dry matter intake; OM, organic matter; ADF, acid detergent fiber; EE, ether extract; N, nitrogen; NDF, neutral detergent fiber; DOM, OM digestibility; DADF, ADF digestibility; DEE, EE digestibility; DCP, Crude protein digestibility; DNDF, NDF digestibility; VFA, total volatile fatty acids.

Redundancy analysis was also conducted on the relative abundance of rumen bacteria and the DMI, nutrient digestibility, total VFA and CH_4_ (Figure 5A), RDA 1 and RDA 2 accounted for 58.7 and 15.1% of the variation, respectively. In the rumen bacteria community structure, the fecal nutrient levels of RAD 1 and RDA 2 represented 31.8 and 24.2%, respectively (Figure 5B). Emission of CH_4_ was the most important factor related to rumen and fecal bacterial structure (*p* < 0.05; Figures 5A,B; Supplementary Table S4). Butyrate and valerate were negatively correlated with bacterial community structure (*p* < 0.05) in the rumen, while the total VFA and acetate were positively correlated with bacterial community structure in feces (*p* < 0.05). In the rumen bacteria community structure, the DMI, nutrient digestibility, total VFA, and CH_4_ of RAD 1 and RDA 2 represented 20.34 and 10.79% (Figure 5C), the fecal nutrient levels of RAD 1 and RDA 2 represented 18.74 and 15.11% (Figure 5D). Fecal ADF content was negatively correlated with the bacterial community structure in rumen. Fecal EE and OM contents were negatively correlated with bacterial community structure in feces (Supplementary Table S4).

### 3.4. Correlations between DMI, nutrient digestibility, total VFA, and bacterial community diversity in rumen and feces

**Figure 6:**
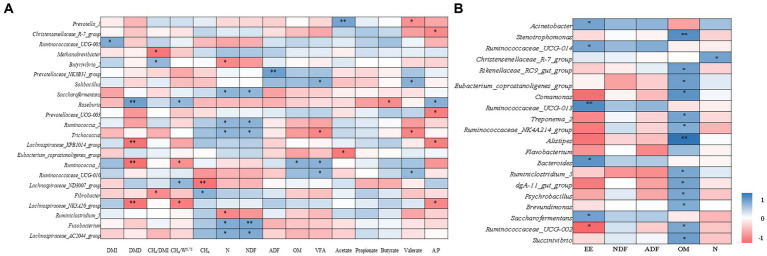
Relationship among DMI, nutrient digestibility, total VFA, CH_4_, and main bacterial communities at genus level in Rumen **(A)**. DMI, dry matter intake; DMD, dry matter digestibility; N, nitrogen; NDF, neutral detergent fiber; ADF, acid detergent fiber; OM, organic matter; VFA, total volatile fatty acids; A:P, acetate to propionate ratio. Relationship among nutrient and main bacterial communities at genus level in feces **(B)**. EE, fecal ether extract; NDF, fecal neutral detergent fiber; ADF, fecal acid detergent fiber; OM, fecal organic matter; N, fecal nitrogen. ** and * indicate significance levels at 0.01 and 0.05, respectively.

In the rumen, DMI was positively correlated with the genera *Ruminococcaceae_UCG-005* (*r* = 0.620) (Figure 6A). Dry matter digestibility was positively correlated with genera *Roseburia* (*r* = 0.699) and negatively correlated with *Lachnospiraceae_XPB1014_group* (*r* = −0.663), *Ruminococcus_1* (*r* = −0.669), and *Lachnospiraceae_NK3A20_group* (*r* = −0.652). Daily CH_4_ emission was positively correlated with genera *Fusobacterium* (*r* = 0.563) and negatively correlated with *Lachnospiraceae_ND3007_group* (*r* = 0.536). The CH_4_/DMI ratio was positively correlated with the genera *Butyrivibrio_2* (*r* = 0.529) and negatively correlated with *Methanobrevibacter* (*r* = −0.638) and *Fibrobacter* (*r* = −0.540). The CH_4_/BW^0.75^ was positively correlated with genera *Roseburia* (*r* = 0.527) and *Lachnospiraceae_ND3007_group* (*r* = 0.536) and negatively correlated with *Ruminococcus_1* (*r* = −0.517) and *Lachnospiraceae_NK3A20_group* (*r* = −0.607). Nitrogen digestibility was positively correlated with genera *Saccharofermentans* (*r* = 0.551), *Ruminococcus_2* (*r* = 0.540), *Trichococcus* (*r* = 0.539), *Fusobacterium* (*r* = 0.611) and *Lachnospiraceae_AC2044_group* (*r* = 0.598) and negatively correlated with *Butyrivibrio_2* (*r* = −0.556) and *Ruminiclostridium_5* (*r* = −0.563). The NDF digestibility was positively correlated with the genera *Saccharofermentans* (*r* = 0.538), *Ruminococcus_2* (*r* = 0.565), *Trichococcus* (*r* = 0.532), *Fusobacterium* (*r* = 0.644) and *Lachnospiraceae_AC2044_group* (*r* = 0.627). Whereas OM digestibility was positively correlated with genera *Ruminococcus_1* (*r* = 0.570). The total VFA concentration was positively correlated with genera *Solibacillus* (*r* = 0.577), *Ruminococcus_1* (*r* = 0.566), and *Ruminococcaceae_UCG-010* (*r* = 0.540) and negatively correlated with *Trichococcus* (*r* = −0.566). Acetate was positively correlated with genera *Prevotella_1* (*r* = 0.667) and negatively correlated with *Eubacterium_coprostanoligenes_group* (*r* = −0.626). Butyrate was negatively correlated with genus *Roseburia* (*r* = −0.547). Valerate was positively correlated with genera *Solibacillus* (*r* = 0.584) and *Ruminococcaceae_UCG-010* (*r* = 0.515) and negatively correlated with *Prevotella_1* (*r* = −0.523) and *Trichococcus* (*r* = −0.580). The acetate to propionate ratio was positively correlated with *Roseburia* (*r* = 0.582) and negatively correlated with *Christensenellaceae_R-7_group* (*r* = −0.553), *Prevotellaceae_UCG-003* (*r* = −0.609), *Lachnospiraceae_XPB1014_group* (*r* = 0.603), and *Lachnospiraceae_NK3A20_group* (*r* = 0.568) genera.

In feces, the EE content was positively correlated with genera *Acinetobacter* (*r* = 0.400), *Ruminococcaceae_UCG-014* (*r* = 0.393), *Ruminococcaceae_UCG-013* (*r* = 0.494), *Bacteroides* (*r* = 0.464), and *Saccharofermentans* (*r* = 0.410), and negatively correlated with *Ruminococcaceae_UCG-002* (*r* = −0.367; Figure 6B). The OM content was positively correlated with the genera *Stenotrophomonas* (*r* = 0.483), *Rikenellaceae_RC9_gut_group* (*r* = 0.417), *Eubacterium_coprostanoligenes_group* (*r* = 0.417), *Comamonas* (*r* = 0.467), *Treponema_2* (*r* = 0.400), *Ruminococcaceae_NK4A214_group* (*r* = 0.367), *Alistipes* (*r* = 0.583), *Ruminiclostridium_5* (*r* = 0.383), *dgA-11_gut_group* (*r* = 0.400), *Psychrobacillus* (*r* = 0.383), and *Brevundimonas* (*r* = 0.367). The N content was positively correlated with the genus *Christensenellaceae_R-7_group* (*r* = 0.407).

### 3.5. Network analysis of bacterial communities in rumen and feces

**Figure 7:**
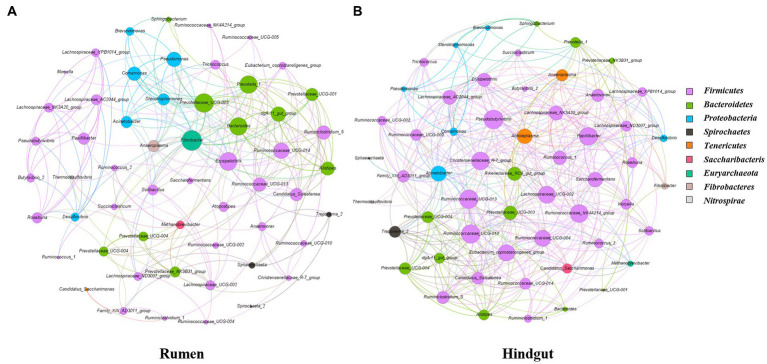
Interaction network of the rumen **(A)** and feces **(B)** microbiota. 16S rRNA gene-based correlation network of the rumen microbiota, displaying statistically significant interactions with absolute value of correlation coefficients >0.6. Node be colored by phylum. The node size was scaled based on the overall abundance of each taxa in the microbiota.

Microbial interactions between rumen and fecal bacterial communities were analyzed using microbial networks (Figure 7). Positive correlation within ruminal bacteria accounted for 68.2 and of the feces accounted for 79% of the total correlation. The putative drivers of keystone taxa in rumen and fecal microbial communities of Simmental calves from the gastrointestinal region were estimated through use of the combined score of the high mean degree, high closeness centrality, and low betweenness centrality (Supplementary Table S5). *Prevotella_1* and *Prevotellaceae_UCG-003* in rumen and *Ruminococcaceae_NK4A214_group*, *Christensenellaceae_R-7_group* and *Ruminococcaceae_UCG-010* in feces could be considered as keystone taxa in Simmental calves.

## 4. Discussion

**Figure 8:**
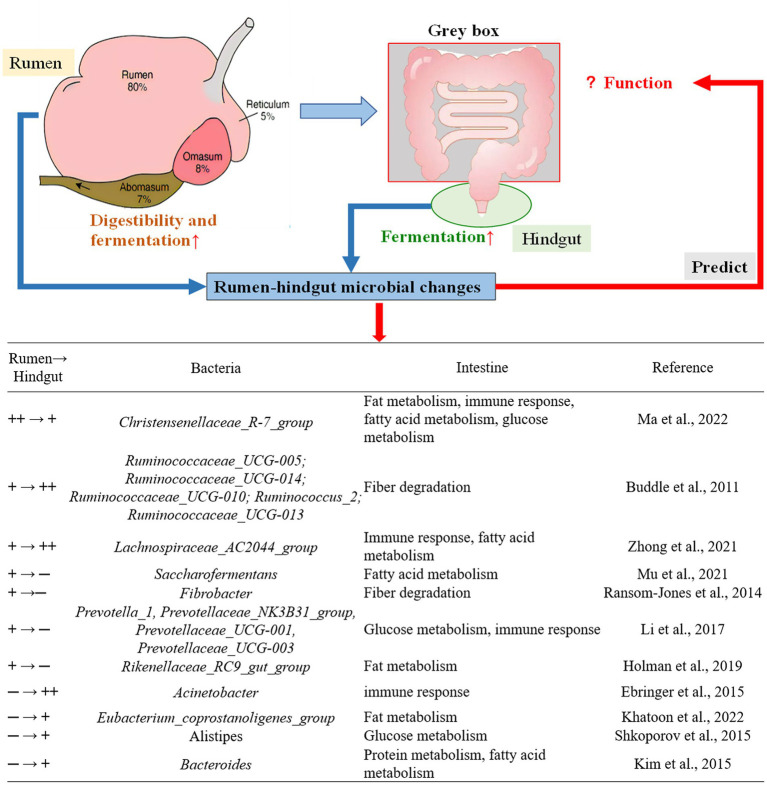
Rumen-hindgut microbial differences predict the corresponding function of small intestine. ++→+, The relative abundance of microorganisms in rumen was greater than in feces (*p* < 0.001); +→++, the relative abundance of microorganisms in feces was greater than in rumen (*p* < 0.05); +→─, microbes in the rumen only; ─→+, microbes in feces only. According to published studies of microbiota change and intestinal function, intestinal function was predicted by rumen-hindgut bacterial differences at the genus level. Gray box, intestinal function was predicted by analyzing rumen-hindgut intestinal changes.

Extracts and other by-products of *A. mongolicum* have the potential to improve average daily gain, feed efficiency, and body antioxidant properties in ruminant species (Panthee et al., 2017; Redoy et al., 2020; Xie et al., 2020). To identify the effect of *A. mongolicum* on the gastrointestinal tract of ruminants, following analyses are necessary for a deep understanding: (1) relationship of microbe functions between rumen and hindgut; (2) the integrated effects to gastrointestinal, and (3) exploring the “gray box” of the intestine through analysis of the microbe in rumen and feces (Figure 8).

The rumen bacteriome supplemented with *A. mongolicum* showed significantly clear separations (Figure 2). Furthermore, significant differences in the bacterial communities between the supplemented and non-supplemented *A. mongolicum* treatments were related to nutrient digestibility (Coasta et al., 2012). Firmicutes and Bacteroidetes constituted the predominant bacteria in rumen and feces, whether *A. mongolicum* supplement was supplemented or not (Figure 3). Firmicutes, which degrade cellulose to total VFAs, constituted the main bacterial type. This bacterium has also been implicated in host immune responses, inhibiting the invasion of opportunistic pathogens, and preventing of inflammation (Gruninger et al., 2014; Zhang et al., 2015) of the intestinal tract. Different regions of the gastrointestinal tract have different effects on the body, facilitating feed energy conversion and harvesting (Chen et al., 2015), and indirectly promoting the physiological function of digestion in calves. Dietary supplementation with *A. mongolicum* promoted feedback mechanism of physiological metabolism through the regulation of Firmicutes, thereby increasing feed intake and ultimately resulting in a decreased proportion of FNH in the DMI (Xie et al., 2020). Supplementation of *A. mongolicum* significantly increased average daily gain of calves (Xie et al., 2020), probably because the ratio of Bacteroidetes to Firmicutes in the rumen tended to be increased by *A. mongolicum* supplementation (*p* = 0.063, Supplementary Table S1), and the ratio of Bacteroidetes to Firmicutes was positively associated with growth rate and feed efficiency (Myer et al., 2015). Therefore, a boosting effect on energy metabolism in cattle was observed. However, the effects of different levels of *A. mongolicum* supplementation on Bacteroidetes were inconsistent. Lower levels (SL200 and SL400) of *A. mongolicum* supplementation did not affect Bacteroidetes significantly; however, SL800 significantly decreased the expansion of Bacteroidetes. This suggested that a critical dose of *A. mongolicum* must be achieved to affect the relative abundance of Bacteroidetes in rumen. It is possible that elevated levels of *A. mongolicum* containing higher concentrations of unsaturated fatty acids may be harmful to the Bacteroidetes (Pitta et al., 2018). The low abundance of the Bacteroidetes may have been compensated through the higher abundance of Firmicutes (Matthews et al., 2019). There were more Bacteroides in feces than in rumen, possibly playing a role in degrading fiber from the rumen in cattle feces (Kim et al., 2015). Thus, it is possible that *A. mongolicum* modulated the relative abundance of Firmicutes and Bacteroidetes to maintain a stable rumen environment, which maintained intestinal stability via balancing the *Acinetobacter* populations in feces. These may be “combined effects” of multiple secondary metabolites of *A. mongolicum*, which can simultaneously regulate Firmicutes and Bacteroidetes in rumen to maintain a healthy gastrointestinal tract.

*Ruminococcaceae* and *Fibrobacter* were well-described genera in the rumen (Figure 4A). The *Ruminococcaceae* was abundant in the rumen and four genera of *Ruminococcaceae* were relatively abundant in fecal microflora (Figure 4B). More efficient ruminants had greater bacterial richness and diversity in their guts, which resulted in more fiber-degrading bacteria in their rumen (Welch et al., 2020). *Ruminococcaceae* is the best-known fiber-degrading bacteria (Biddle et al., 2013), and the relative abundance of *Ruminococcaceae_UCG-005* in SL400 was increased significantly. This observation suggested that supplementation of *A. mongolicum* may improve the feed conversion efficiency of Simmental calves. *Ruminococcaceae* also acted synergistically with other rumen microorganisms (Bloemendaal et al., 2021). For example, the relative abundance of *Fibrobacter* was greater in rumen than in feces, which may be the main rumen fibrinolytic bacteria that can decompose cellulose and pentosan into usable organic acids (acetate, propionate, butyrate, etc.; Ransom-Jones et al., 2012). An increase in these organic acids (acetate, propionate, butyrate, etc) were associated with negative effects on fibrinolytic bacteria and biohydrogenation. A reduction in biohydrogenation leads to greater flow of organic acids to the gut. Thus, absorbing and storing these fatty acids in adipose tissue as body fat (Ransom-Jones et al., 2014) and indirectly promoting intestinal lipid metabolism. CH_4_ emission was the most important factor related to rumen and fecal bacterial structure (Figure 5). *Fibrobacter* in the rumen was positively correlated with CH_4_ emissions (Figure 6A), and the main metabolite of fiber degradation was hydrogen, which ultimately increased the hydrogen ions required for the process of CH_4_ production (Xie et al., 2022). The genus *Ruminococcus* in rumen was positively correlated with NDF and OM digestibility (Figure 6A), thus promoting sufficient degradation of fiber in the rumen (Firkins, 2021).

Although the genera *Prevotella* and *Alistipes* were commonly found in cattle feces, their relative abundance were more significantly affected by the level *A. mongolicum* supplementation and the region of the gut sampled. *Prevotella_1* was a potential biomarker for individualized nutritional management of obesity. Decrease in the relative abundance of *Prevotella_1* enhanced the immune performance of ruminant by reducing local and systemic diseases (Xie et al., 2020). Similar results due to *A. mongolicum* supplementation have already been reported (Kholy et al., 2015). To further support this hypothesis, it has been reported that *A. mongolicum* supplementation inhibited the body’s metabolism by reducing the abundance of *Prevotella_1* in rumen, thereby increasing the daily gain of ruminants (Tett et al., 2021). Flavonoids in *A. mongolicum* can promote the decomposition of plant polysaccharides by increasing the relative abundance of *Prevotella*, while most complex plant polysaccharides from diet are not digested by livestock and enter the colon as a potential nutrient source for the microbiota (Briggs et al., 2020; Ghimire et al., 2022), therefore, *A. mongolicum* supplementation increased the relative abundance of *Prevotella* in feces in SL800 group. *Alistipes* are often glycolytic (Shkoporov et al., 2015), which may explain their association with hindgut rather than with rumen.

*Acinetobacter* is a conditioned pathogen, so the relative abundance of *Acinetobacter* in feces may have been reduced due to the antibacterial activity of flavonoids in *A. mongolicum*, which may have required a critical mass dosage of *A. mongolicum* (SL400 and SL800) to exert such effects. *A. mongolicum* supplementation maintains the stability of the intestinal flora (Klevenhusen et al., 2011; Ebringer et al., 2015). Conversely, if the number of pathogenic bacteria can be reduced, and therefore less excreted in feces, the infectious environmental pollution can be reduced (Wilson et al., 2020). Antioxidant index (superoxide dismutase and glutathione peroxidase) in serum were improved in Simmental calves given SL400 (Xie et al., 2020), which may be due to the increased relative abundance of Actinobacteria (Supplementary Table S2), thereby helping to maintain intestinal barrier functions, increasing tight junctions’ expression, regulating mucin biosynthesis and catabolism, providing energy for epithelial cells proliferation and stimulating the immune system (D’Argenio and Salvatore, 2015; Zhu et al., 2016). *Rikenellaceae_RC9_gut_group* abundance was significantly higher in SL800, which may play an important role in rumen lipid metabolism (Holman and Gzyl, 2019), thereby promoting the role of *A. mongolicum* in intestinal metabolism. Additionally, *A. mongolicum* supplementation could contribute to glucose metabolism to produce acetate and butyrate via *Rikenellaceae_RC9_gut_group* (Seshadri et al., 2018). A large amount of cellulose can be converted into propionic acid by the action of *Rikenellaceae_R9_gut_group* in the digestive tract of ruminants. The relative abundance of *Rikenellaceae_R9_gut_group* in SL800 was increased in feces, thereby increasing the propionic concentration to promote gluconeogenesis in Simmental calves (Aschenbach et al., 2010; Graf, 2014). Thus, in this study, the regulation mechanism of intestine microbiota in the metabolism of glucose, protein, amino acid, as well as lipid metabolism were explored by analyzing the microbial community structure of the rumen and feces and their corresponding functions.

The important role of the gastrointestinal microbiota in intestinal function and animal production has been clarified. This study identified a trapezoid taxon with network topological properties in the gastrointestinal tract of Simmental cattle (Figure 7). The balance and stability of microbial community in the gastrointestinal tract contribute to host health, with key genera differing between the rumen and feces. Network analyses can reveal interactions between species (Liu H. et al., 2020). Both rumen and fecal microbes had higher of positive correlations than negative correlations. The positive interactions might strengthen and negative interaction might weaken competitive (Fan et al., 2021). Microbes in the rumen were more positively correlated than in feces. It is reasonable to consider that the rumen is a unique digestive chamber of the gut for digestion, absorption and associated immune responses. In on-farm greenhouse gas emissions models, microorganisms can make full use of limited feed resources by strengthening cooperation (Vibart et al., 2021). The main function of the back end of the digestive tract was digestion, decomposition and absorption, which contains a large number of enzymes and microorganisms.

## 5. Conclusion

Dietary supplementation with *A. mongolicum* altered the rumen and fecal microflora of Simmental calves, which positively affected microbial community structure in rumen and feces. The microbiome diversity of rumen and feces significantly affected under the SL400 and SL800 treatments, but not under SL200. The addition of *A. mongolicum* increased relative abundance *Rikenellaceae_RC9_gut_group* and *Ruminococcaceae_UCG-010* in rumen, and decreased relative abundance of Bacteroidetes and *Prevotella_1* in rumen, which enhanced diet digestibility and fermentation. Both the increase of Firmicutes, *Prevotella_1, Ruminococcaceae_UCG-005 Christensenellaceae_R-7_group* abundance, and the decrease of *Acinetobacter, Ruminiclostridium_5,* and *Ruminococcaceae_UCG-014* abundance could promote fecal fermentation with a positive effect on Simmental calf health. This study demonstrated that rumen-hindgut microbial were affected by supplementation of a certain amount of *A. mongolicum*, further studies are needed to determine the mechanisms of observed effects.

## Data availability statement

The data presented in this study are deposited in the NCBI repository, accession number PRJNA941479.

## Ethics statement

All animal experimental procedures were authorized by the Laboratory Animal Management and Welfare Committee of Lanzhou University (file No: 2010-1 and 2010-2).

## Author contributions

FH: conceptualization, supervision, and experimental design. KX: data analysis and original draft writing. KX, YG, and CZ: animal feeding experiment. JN and CZ: laboratory sample analyses. FH, SC, and TY: review and editing. All authors contributed to the article and approved the submitted version.

## Funding

FH acknowledges the scholarship by the National Natural Science Foundation of China (32161143028); the Program of National Science and Technology Assistance (KY202002011); the Lanzhou City’s Scientifc Research Funding Subsidy to Lanzhou University; and the Program for Innovative Research Team of Ministry of Education (IRT17R50).

## Conflict of interest

The authors declare that the research was conducted in the absence of any commercial or financial relationships that could be construed as a potential conflict of interest.

## Publisher’s note

All claims expressed in this article are solely those of the authors and do not necessarily represent those of their affiliated organizations, or those of the publisher, the editors and the reviewers. Any product that may be evaluated in this article, or claim that may be made by its manufacturer, is not guaranteed or endorsed by the publisher.

## Supplementary material

The Supplementary material for this article can be found online at: https://www.frontiersin.org/articles/10.3389/fmicb.2023.1174740/full#supplementary-material

## Footnotes

^1^ http://ccb.jhu.edu/software/FLASH/

^2^ http://qiime.org/scripts/split_libraries_fastq.html

^3^ http://www.drive5.com/usearch/manual/uchime_algo.html

^4^ http://drive5.com/uchime/uchime_download.html

^5^ https://CRAN.R-project.org

^6^ https://gephi.org
