# Supplementary material for: Dietary supplementation of Allium mongolicum modulates rumen-hindgut microbial community structure in Simmental calves
Source: Front Microbiol. 2023 Jun 7;14:1174740. doi: 10.3389/fmicb.2023.1174740 (PMC10284144; doi:10.3389/fmicb.2023.1174740)
Supplement: Supplementary file 1 [file Table_1.DOCX]

Supplementary Material

Dietary supplementation of *Allium mongolicum* modulates rumen-hindgut microbial community structure in Simmental calves

**Kaili Xie^1^, Shenghua Chang^1^; Jiao Ning^1^; Yarong Guo^1^; Cheng Zhang^1^; Tianhai Yan^2^; Fujiang Hou^1^***

*** Correspondence:** Fujiang Hou: [cyhoufj@lzu.edu.cn](mailto:cyhoufj@lzu.edu.cn)

# Supplementary Tables

# Table S1 Composition of rumen bacterial communities in Simmental calves at phylum and genus levels (only taxa with an average relative abundance > 0.5%).

| Items | Treatments | | | | SEM | *P* |
| --- | --- | --- | --- | --- | --- | --- |
|  | SL0 | SL200 | SL400 | SL800 |  |  |
| Phylum |  |  |  |  |  |  |
| Firmicutes | 51.02 | 56.64 | 54.05 | 57.89 | 1.323 | 0.159 |
| Proteobacteria | 0.88^b^ | 1.09^b^ | 0.75^b^ | 2.04^a^ | 0.186 | 0.034 |
| Bacteroidetes | 45.10^a^ | 38.04^ab^ | 41.81^ab^ | 36.34^b^ | 1.414 | 0.026 |
| Spirochaetes | 0.51 | 1.51 | 0.40 | 0.47 | 0.270 | 0.639 |
| Tenericutes | 0.74 | 0.81 | 0.71 | 0.90 | 0.043 | 0.325 |
| Firmicutes/bacteroidetes | 1.15 | 1.54 | 1.30 | 1.64 | 0.088 | 0.063 |
| Genus |  |  |  |  |  |  |
| *Prevotella_1* | 27.00^a^ | 16.83^b^ | 21.53^ab^ | 14.74^b^ | 1.592 | 0.021 |
| *Christensenellaceae_R-7_group* | 13.4 | 12.98 | 14.22 | 15.25 | 0.69 | 0.340 |
| *Rikenellaceae_RC9_gut_group* | 7.31^b^ | 7.22^b^ | 6.30^b^ | 11.73^a^ | 0.725 | 0.009 |
| *Ruminococcaceae_NK4A214_group* | 6.69 | 7.46 | 7.26 | 7.69 | 0.196 | 0.150 |
| *Prevotellaceae_NK3B31_group* | 0.5 | 0.76 | 1.61 | 0.52 | 0.252 | 0.703 |
| *Treponema_2* | 0.41 | 1.41 | 0.34 | 0.39 | 0.27 | 0.664 |
| *Saccharofermentans* | 2.33 | 2.88 | 2.32 | 2.42 | 0.114 | 0.785 |
| *Prevotellaceae_UCG-001* | 1.61 | 1.158 | 1.30 | 1.07 | 0.725 | 0.230 |
| *Prevotellaceae_UCG-003* | 1.85 | 1.07 | 2.30 | 1.06 | 0.228 | 0.545 |
| *Ruminococcaceae_UCG-014* | 1.24^b^ | 1.80^ab^ | 1.14^b^ | 1.57^ab^ | 0.098 | 0.657 |
| *Ruminococcus_2* | 0.566 | 0.685 | 0.639 | 0.548 | 0.079 | 0.903 |
| *Ruminococcaceae_UCG-010* | 1.20^b^ | 1.50^a^ | 1.42^a^ | 1.46^a^ | 0.061 | 0.241 |
| *Lachnospiraceae_NK3A20_group* | 0.668 | 0.556 | 0.566 | 0.923 | 0.076 | 0.274 |

SL0, 0 mg/kg BW *A. mongolicum*; SL200, 200 mg/kg BW *A. mongolicum*; SL400, 400 mg/kg BW *A. mongolicum*; SL800, 800 mg/kg BW *A. mongolicum*.

^a,b^ Different letters represent significant differences in the rows (*p* < 0.05); SEM, standard error of mean.

# Table S2 Composition of fecal bacterial communities in Simmental calves at phylum and genus levels (only taxa with an average relative abundance > 0.5%).

| Items | Treatments | | | | SEM | *P* |
| --- | --- | --- | --- | --- | --- | --- |
|  | SL0 | SL200 | SL400 | SL800 |  |  |
| Phylum |  |  |  |  |  |  |
| Firmicutes | 56.23^b^ | 47.64^b^ | 66.59^a^ | 60.43^a^ | 4.355 | 0.004 |
| Proteobacteria | 20.37 | 40.68 | 13.55 | 15.20 | 5.837 | 0.954 |
| Bacteroidetes | 18.64 | 10.12 | 15.44 | 22.08 | 2.087 | 0.466 |
| Spirochaetes | 1.19^a^ | 0.23^b^ | 2.12^a^ | 0.34^b^ | 0.392 | 0.005 |
| Tenericutes | 1.91^a^ | 0.56^b^ | 0.58^b^ | 0.61^b^ | 0.173 | 0.003 |
| Actinobacteria | 0.14^b^ | 0.31^ab^ | 0.57^a^ | 0.39^ab^ | 0.057 | 0.038 |
| Firmicutes/bacteroidetes | 3.37 | 4.85 | 4.68 | 3.09 | 0.415 | 0.365 |
| Genus |  |  |  |  |  |  |
| *Acinetobacter* | 18.09^a^ | 16.48^a^ | 9.50^b^ | 3.93^b^ | 2.105 | 0.017 |
| *Solibacillus* | 2.4 | 8.62 | 10.48 | 11.55 | 1.807 | 0.193 |
| *Prevotella_1* | 0.62^b^ | 0.66^b^ | 0.58^b^ | 6.02^a^ | 0.098 | 0.005 |
| *Atopostipes* | 0.82 | 5.85 | 7.07 | 1.49 | 0.262 | 0.086 |
| *Ruminococcaceae_UCG-005* | 3.01^b^ | 1.30^ab^ | 10.14^a^ | 8.40^ab^ | 1.442 | 0.014 |
| *Stenotrophomonas* | 0.26^b^ | 0.66^b^ | 0.35^b^ | 4.05^a^ | 0.184 | 0.006 |
| *Ruminococcaceae_UCG-014* | 6.96^a^ | 1.66^b^ | 2.62^b^ | 1.94^b^ | 1.401 | 0.004 |
| *Trichococcus* | 0.63^b^ | 3.74^a^ | 0.19^b^ | 0.20^b^ | 0.048 | 0.006 |
| *Christensenellaceae_R-7_group* | 2.99^b^ | 2.03^b^ | 4.23^ab^ | 5.42^a^ | 0.621 | 0.041 |
| *Rikenellaceae_RC9_gut_group* | 5.22 | 3.42 | 5.43 | 5.89 | 0.686 | 0.638 |
| *Eubacterium_coprostanoligenes_group* | 6.00^a^ | 1.96^b^ | 2.43^b^ | 2.37^b^ | 0.606 | 0.013 |
| *Ruminococcaceae_UCG-013* | 4.12^a^ | 0.70^b^ | 2.77^ab^ | 1.86^ab^ | 0.521 | 0.075 |
| *Ruminococcaceae_NK4A214_group* | 3.11 | 1.82 | 2.09 | 2.69 | 0.291 | 0.457 |
| *Ruminococcaceae_UCG-010* | 3.55^a^ | 1.17^ab^ | 2.37^b^ | 2.17^ab^ | 0.354 | 0.096 |
| *Alistipes* | 2.97^a^ | 0.67^b^ | 0.96^b^ | 0.73^b^ | 0.315 | 0.017 |
| *Bacteroides* | 1.84 | 1.08 | 2.28 | 1.385 | 0.284 | 0.254 |
| *Prevotellaceae_UCG-003* | 1.74 | 0.57 | 1.31 | 0.995 | 0.244 | 0.216 |
| *Ruminiclostridium_5* | 2.21^a^ | 0.55^b^ | 0.83^b^ | 0.75^b^ | 0.224 | 0.009 |
| *dgA-11_gut_group* | 1.93^a^ | 0.51^b^ | 0.64^b^ | 0.45^b^ | 0.210 | 0.001 |
| *Prevotellaceae_UCG-004* | 1.13 | 0.66 | 1.34 | 0.79 | 0.204 | 0.621 |
| *Lachnospiraceae_AC2044_group* | 0.67 | 0.43 | 1.34 | 1.13 | 0.158 | 0.187 |

SL0, 0 mg/kg BW *A. mongolicum*; SL200, 200 mg/kg BW *A. mongolicum*; SL400, 400 mg/kg BW *A. mongolicum*; SL800, 800 mg/kg BW *A. mongolicum*.

^a,b^ Different letters represent significant differences in the rows (*p* < 0.05); SEM, standard error of mean.

# Table S3 Effect of *A. mongolicum* on the rumen and fecal microorganisms of Simmental calves at the genus level (only taxa with an average relative abundance > 0.5%).

| Items | Regions | Treatment | Regions *Treatment |
| --- | --- | --- | --- |
| *Acinetobacter* | 0.001 | 0.079 | 0.070 |
| *Prevotella_1* | <0.001 | 0.356 | 0.025 |
| *Christensenellaceae_R-7_group* | <0.001 | 0.173 | 0.973 |
| *Rikenellaceae_RC9_gut_group* | 0.002 | 0.038 | 0.216 |
| *Ruminococcaceae_UCG-005* | 0.046 | 0.169 | 0.019 |
| *Solibacillus* | 0.011 | 0.726 | 0.738 |
| *Ruminococcaceae_UCG-014* | 0.008 | 0.059 | 0.023 |
| *Eubacterium_coprostanoligenes_group* | 0.011 | 0.089 | 0.041 |
| *Ruminococcaceae_UCG-010* | 0.009 | 0.212 | 0.073 |
| *Ruminiclostridium_5* | <0.001 | 0.038 | 0.01 |
| *Prevotellaceae_NK3B31_group* | 0.005 | 0.399 | 0.327 |
| *Saccharofermentans* | <0.001 | 0.597 | 0.213 |
| *Prevotellaceae_UCG-001* | <0.001 | 0.743 | 0.332 |
| *Fibrobacter* | <0.001 | 0.872 | 0.779 |
| *Lachnospiraceae_NK3A20_group* | <0.001 | 0.132 | 0.692 |
| *Ruminococcaceae_UCG-013* | <0.001 | 0.155 | 0.080 |
| *Ruminococcaceae_NK4A214_group* | <0.001 | 0.649 | 0.270 |
| *Alistipes* | <0.001 | 0.016 | 0.009 |
| *Bacteroides* | <0.001 | 0.535 | 0.419 |
| *dgA-11_gut_group* | <0.001 | 0.030 | 0.018 |
| *Prevotellaceae_UCG-004* | 0.005 | 0.588 | 0.726 |

# Table S4 Mantel test of the microbial community with nutrient digestibility, rumen fermentation parameters, and fecal nutrients in different treatments

| Parameters | Rumen | | Hindgut | |
| --- | --- | --- | --- | --- |
|  | R | *P* | R | *P* |
| DMI | -0.389 | 0.342 | -0.5 | 0.153 |
| DCP | -0.519 | 0.131 | -0.354 | 0.419 |
| DNDF | 0.169 | 0.837 | -0.101 | 0.931 |
| DADF | 0.253 | 0.66 | 0.199 | 0.756 |
| DOM | 0.455 | 0.223 | -0.286 | 0.585 |
| CH_4_ | -0.761 | 0.008** | -0.640 | 0.030* |
| VFA | -0.588 | 0.067 | 0.665 | 0.014* |
| Acetate | -0.484 | 0.185 | 0.635 | 0.027* |
| Propionate | -0.376 | 0.375 | -0.546 | 0.095 |
| Butyrate | -0.641 | 0.031* | -0.523 | 0.131 |
| Valerate | -0.632 | 0.041* | -0.512 | 0.132 |
| Fecal nutrient content |  |  |  |  |
| EE | 0.79 | 0.107 | -0.540 | <0.001** |
| NDF | 0.291 | 0.075 | -0.582 | 0.574 |
| ADF | -0.180 | 0.014* | 0.699 | 0.809 |
| OM | 0.643 | 0.128 | -0.509 | 0.026* |
| N | 0.422 | 0.885 | -0.136 | 0.277 |

# DMI=dry matter intake; DMD=dry matter digestibility; DCP=crud protein digestibility; DNDF=neutral detergent fiber digestibility; DADF=acid detergent fiber digestibility; DOM=organic matter digestibility; VFA=volatile fatty acids

# Table S5. Comparison of Degree, Closness centrality, and Betweenness centrality of Simmental calf in the rumen and feces.

| Rumen | | | | Hindgut | | | | |
| --- | --- | --- | --- | --- | --- | --- | --- | --- |
| Id | Degree | Closness centrality | Betweeness centrality | | Id | Degree | Closness centrality | Betweeness centrality |
| *Prevotella_1* | 16 | 0.458333 | 45.467774 | | *Prevotella_1* | 14 | 0.504587 | 75.915848 |
| *Christensenellaceae_R-7_group* | 4 | 0.359477 | 27.402183 | | *Christensenellaceae_R-7_group* | 26 | 0.639535 | 76.444648 |
| *Ruminococcaceae_NK4A214_group* | 3 | 0.339506 | 13.188448 | | *Rikenellaceae_RC9_gut_group* | 19 | 0.572917 | 21.085392 |
| *Acinetobacter* | 10 | 0.436508 | 50.393571 | | *Ruminococcaceae_NK4A214_group* | 26 | 0.617978 | 83.323381 |
| *Ruminococcaceae_UCG-005* | 3 | 0.292553 | 54 | | *Acinetobacter* | 22 | 0.604396 | 89.581411 |
| *Butyrivibrio_2* | 7 | 0.323529 | 0 | | *Ruminococcaceae_UCG-005* | 15 | 0.539216 | 8.493585 |
| *Saccharofermentans* | 5 | 0.348101 | 58.191667 | | *Butyrivibrio_2* | 6 | 0.413534 | 0.933122 |
| *Eubacterium_coprostanoligenes_group* | 6 | 0.40146 | 41.647095 | | *Saccharofermentans* | 21 | 0.585106 | 43.673573 |
| *Prevotellaceae_UCG-003* | 17 | 0.482456 | 61.364912 | | *Eubacterium_coprostanoligenes_group* | 24 | 0.625 | 56.406671 |
| *Ruminococcaceae_UCG-014* | 14 | 0.44 | 15.551746 | | *Prevotellaceae_UCG-003* | 19 | 0.572917 | 38.340384 |
| *Ruminococcaceae_UCG-010* | 5 | 0.384615 | 34.090614 | | *Ruminococcaceae_UCG-014* | 16 | 0.528846 | 12.258627 |
| *Prevotellaceae_UCG-001* | 11 | 0.419847 | 11.874564 | | *Ruminococcaceae_UCG-010* | 27 | 0.625 | 47.886541 |
| *Lachnospiraceae_XPB1014_group* | 7 | 0.323529 | 0 | | *Prevotellaceae_UCG-001* | 2 | 0.345912 | 0 |
| *Ruminococcus_1* | 2 | 0.341615 | 0 | | *Lachnospiraceae_XPB1014_group* | 12 | 0.5 | 7.038794 |
| *Prevotellaceae_NK3B31_group* | 9 | 0.416667 | 100.75587 | | *Ruminococcus_1* | 19 | 0.591398 | 19.589182 |
| *Lachnospiraceae_AC2044_group* | 9 | 0.357143 | 27.710056 | | *Prevotellaceae_NK3B31_group* | 7 | 0.478261 | 2.140401 |
| *Lachnospiraceae_ND3007_group* | 7 | 0.433071 | 76.172003 | | *Lachnospiraceae_AC2044_group* | 20 | 0.591398 | 18.174933 |
| *Pseudobutyrivibrio* | 7 | 0.323529 | 0 | | *Lachnospiraceae_ND3007_group* | 14 | 0.518868 | 11.340701 |
| *Lachnospiraceae_NK3A20_group* | 8 | 0.381944 | 61.58239 | | *Pseudobutyrivibrio* | 28 | 0.662651 | 94.231803 |
| *Anaerovorax* | 7 | 0.384615 | 76.153247 | | *Lachnospiraceae_NK3A20_group* | 20 | 0.597826 | 77.455201 |
| *Papillibacter* | 10 | 0.410448 | 95.345281 | | *Anaerovorax* | 17 | 0.561224 | 19.575582 |
| *Methanobrevibacter* | 7 | 0.376712 | 56.043216 | | *Papillibacter* | 26 | 0.639535 | 81.446132 |
| *Ruminococcus_2* | 5 | 0.352564 | 12.170763 | | *Methanobrevibacter* | 7 | 0.482456 | 0.463433 |
| *Succiniclasticum* | 5 | 0.390071 | 48.985233 | | *Ruminococcus_2* | 12 | 0.509259 | 9.856537 |
| *Solibacillus* | 9 | 0.429688 | 25.640096 | | *Succiniclasticum* | 8 | 0.470085 | 9.322884 |
| *Roseburia* | 9 | 0.381944 | 92.86129 | | *Solibacillus* | 12 | 0.504587 | 4.656736 |
| *Ruminococcaceae_UCG-013* | 12 | 0.458333 | 71.372828 | | *Roseburia* | 14 | 0.52381 | 8.483193 |
| *Treponema_2* | 4 | 0.395683 | 73.198617 | | *Ruminococcaceae_UCG-013* | 27 | 0.625 | 48.061612 |
| *Prevotellaceae_UCG-004* | 8 | 0.470085 | 97.016712 | | *Treponema_2* | 16 | 0.55 | 12.703346 |
| *Family_XIII_AD3011_group* | 6 | 0.40146 | 95.204956 | | *Prevotellaceae_UCG-004* | 16 | 0.5 | 14.951759 |
| *Ruminiclostridium_5* | 12 | 0.429688 | 45.38415 | | *Family_XIII_AD3011_group* | 16 | 0.539216 | 24.551564 |
| *Trichococcus* | 8 | 0.426357 | 11.536904 | | *Ruminiclostridium_5* | 17 | 0.52381 | 19.372517 |
| *Moryella* | 1 | 0.227273 | 0 | | *Trichococcus* | 11 | 0.5 | 41.060381 |
| *Ruminococcaceae_UCG-002* | 5 | 0.369128 | 80.855223 | | *Moryella* | 14 | 0.514019 | 14.492282 |
| *Ruminococcaceae_UCG-004* | 3 | 0.361842 | 14.325529 | | *Ruminococcaceae_UCG-002* | 12 | 0.518868 | 4.74576 |
| *Alistipes* | 12 | 0.413534 | 5.298683 | | *Ruminococcaceae_UCG-004* | 23 | 0.604396 | 47.621762 |
| *Atopostipes* | 8 | 0.413534 | 63.032136 | | *Alistipes* | 14 | 0.486726 | 8.28599 |
| *Bacteroides* | 16 | 0.486726 | 99.895716 | | *Bacteroides* | 5 | 0.381944 | 0 |
| *Ruminiclostridium_1* | 3 | 0.325444 | 13.804762 | | *Ruminiclostridium_1* | 10 | 0.470085 | 2.784993 |
| *Desulfovibrio* | 7 | 0.323529 | 0 | | *Desulfovibrio* | 9 | 0.478261 | 3.444913 |
| *Stenotrophomonas* | 10 | 0.423077 | 12.782866 | | *Stenotrophomonas* | 6 | 0.416667 | 1.739662 |
| *Comamonas* | 12 | 0.474138 | 91.597293 | | *Comamonas* | 10 | 0.518868 | 66.625914 |
| *Brevundimonas* | 7 | 0.395683 | 55.070513 | | *Brevundimonas* | 6 | 0.410448 | 4.29629 |
| *dgA-11_gut_group* | 13 | 0.429688 | 14.527788 | | *dgA-11_gut_group* | 14 | 0.509259 | 23.63185 |
| *Erysipelothrix* | 16 | 0.470085 | 100.32698 | | *Erysipelothrix* | 19 | 0.591398 | 30.845629 |
| *Candidatus_Soleaferrea* | 11 | 0.429688 | 18.895188 | | *Candidatus_Soleaferrea* | 17 | 0.572917 | 26.032408 |
| *Lachnospiraceae_UCG-002* | 8 | 0.423077 | 58.773847 | | *Lachnospiraceae_UCG-002* | 25 | 0.625 | 41.634645 |
| *Prevotellaceae_UCG-004* | 8 | 0.470085 | 97.016712 | | *Prevotellaceae_UCG-004* | 16 | 0.5 | 14.951759 |
| *Sphingobacterium* | 4 | 0.345912 | 0.821614 | | *Sphingobacterium* | 6 | 0.410448 | 4.29629 |
| *Thermodesulfovibrio* | 3 | 0.305556 | 0 | | *Thermodesulfovibrio* | 3 | 0.416667 | 0 |
| *Pseudomonas* | 13 | 0.466102 | 96.776108 | | *Pseudomonas* | 6 | 0.416667 | 1.739662 |
| *Candidatus_Saccharimonas* | 2 | 0.298913 | 0 | | *Candidatus_Saccharimonas* | 13 | 0.504587 | 12.629102 |
| *Anaeroplasma* | 10 | 0.416667 | 15.106324 | | *Acholeplasma* | 21 | 0.585106 | 30.765406 |
| *Fibrobacter* | 18 | 0.482456 | 117.33407 | | *Anaeroplasma* | 16 | 0.555556 | 19.983997 |
| *Sphaerochaeta* | 5 | 0.390071 | 40.452462 | | *Fibrobacter* | 8 | 0.466102 | 21.631811 |
| *Spirochaeta_2* | 1 | 0.284974 | 0 | | *Sphaerochaeta* | 1 | 0.37931 | 0 |
